# Relationship between symptom burden and disability leave among patients with myeloproliferative neoplasms (MPNs): findings from the Living with MPN patient survey

**DOI:** 10.1007/s00277-019-03610-4

**Published:** 2019-01-29

**Authors:** Jingbo Yu, Dilan Paranagama, Holly L. Geyer, Shreekant Parasuraman, Ruben Mesa

**Affiliations:** 1 Incyte Corporation, 1801 Augustine Cut-Off, Wilmington, DE 19803 USA; 2 Mayo Clinical Cancer Center, Scottsdale, AZ USA; 3 UT Health San Antonio Cancer Center, San Antonio, TX USA

**Keywords:** Myeloproliferative neoplasm, Essential thrombocythemia, Polycythemia vera, Myelofibrosis, Disability leave

## Abstract

Patients with myeloproliferative neoplasms (MPNs) experience burdensome symptoms that negatively affect their quality of life. How MPN symptoms relate with medical disability leave (MDL) among patients with the disease has not been previously examined. Using data collected from the Living with MPNs patient survey, symptom burden and functional status were compared in patients who reported taking MDL due to their MPN versus patients who reported no changes in employment status. Among 592 patients who were employed full- or part-time at diagnosis, 24.8% reported taking ≥ 1 MDL and 49.4% reported no change in employment status as a result of their MPN. Of the patients who took MDL, 29.9% took ≥ 2 MDLs, and most patients (62.6%) did not return to work. All 10 symptoms comprising the MPN Symptom Assessment Form were significantly more frequent and severe in patients who took MDL compared with those who had no employment change. Furthermore, functional impairments were also significantly more frequent among patients who went on MDL versus those with no employment change. Effective management of MPN-related symptoms may reduce disability leave among patients with high symptom burden.

## Introduction

Patients with chronic Philadelphia chromosome-negative myeloproliferative neoplasms (MPNs), including myelofibrosis (MF), polycythemia vera (PV), and essential thrombocythemia (ET), have reduced survival compared with the general population [1, 2] and may experience substantial symptom burden during the course of their disease [3, 4]. Commonly reported symptoms include fatigue (45–99%), early satiety (21–77%), inactivity (19–77%), night sweats (25–63%), bone pain (23–55%), and problems with concentration (19–73%) [3–6]. These common symptoms may be severe based on MPN Symptom Assessment Form (MPN-SAF) scores (fatigue, 2.9–6.7; early satiety, 1.0–3.2; inactivity, 1.0–6.7; night sweats, 1.9–2.6; bone pain, 1.2–2.2; and problems with concentration, 1.5–6.1) [3–6]. In the MPN Landmark survey of patients with MF, PV, or ET, many patients reported a decrease in quality of life (MF, 81%; PV, 66%; ET, 57%), and interference with activities of daily living (MF, 53%; PV, 48%; ET, 37%) as a result of their disease [4]. Additionally, several studies have reported that a significant proportion of patients with MPNs require support of a caregiver (40–55%) and many employed patients experience an overall work impairment (31–36%) [6–8].

The effects of MPN symptom burden on patients’ ability to work or employment status were reported in two studies to date. Among employed respondents of the MPN Landmark survey, many reported reduced work hours because of their MPN (MF, 59%; PV, 37%; ET, 30%) [4], suggesting that symptom burden may have a significant impact on employment status. In a previous analysis of the Living with MPNs survey, approximately half (51%) of all employed respondents reported at least one change in employment status (e.g., left job, took early retirement, took disability leave, reduced work hours) as a result of their MPN [7]. Furthermore, more severe symptom burden as assessed by the MPN-SAF total symptom score (TSS) was highly correlated with reduced work productivity as measured with the Work Productivity and Activity Impairment Specific Health Problem questionnaire (correlation coefficients: absenteeism, 0.37; presenteeism, 0.70; work impairment, 0.70; activity impairment, 0.70 [all *P* < 0.001]) [7]. In this analysis, the characteristics of medical disability leave (MDL) among patients with MPNs and the relationship between MDL and MPN symptom burden were examined using data from the Living with MPNs survey.

## Patients and methods

### Study design and participants

The Living with MPNs survey was a cross-sectional online questionnaire conducted between April and November 2016. The study received approval from the Quorum Review Institutional Review Board. Respondents were recruited through emails; online posts on MPN-focused social media and patient advocacy websites; banner advertisements on medical websites, Google, and Facebook; and postcards distributed to the offices of hematologists and oncologists. Survey participants were offered $25 in compensation for completing the survey and provided informed consent electronically before responding to the survey. Respondents were 18 to 70 years of age with a diagnosis of MF, PV, or ET in the USA [7]. The survey was designed to be completed in approximately 30 min and included approximately 100 questions related to patient demographics; MPN diagnosis and disease-related medical history; changes in employment status, work productivity, and daily activities; MPN-related symptoms; functional status; and quality-of-life measures [7]. For those patients who reported being employed full-time or part-time at the time of diagnosis with MF, PV, or ET, a series of questions was asked about various types of employment changes (e.g., left job, took early retirement, took MDL, had reduced work hours, and changed from full-time to part-time job) due to MPN disease and the specifics of those changes. Based on these questions, patients who took MDL after diagnosis because of their MPN disease were identified. For those who indicated that they took any MDL because of their MPN disease, the number of MDLs, most recent MDL start and end year, type (i.e., long-term or short-term), receipt of social security benefits, and whether patients returned to work following the most recent MDL were reported.

The MPN-SAF was used to assess the severity of the 10 most clinically relevant and characteristic MPN symptoms (abdominal discomfort, bone pain, early satiety, fatigue, fever, inactivity, itching, night sweats, problems with concentration, and unintentional weight loss) on a scale from 0 (absent) to 10 (worst) [3]. Symptoms were further categorized by severity based on MPN-SAF score (none, 0; mild, 1–3; moderate, 4–6; severe, ≥ 7). The MPN-SAF TSS, the sum of the 10 individual symptom scores, was also calculated, with maximum TSS of 100 [3]. Additionally, 15 questions on functional status in physical, mental, and social domains were assessed with a five-scale response (“not at all,” “a little bit,” “somewhat,” “quite a bit,” or “a great deal”).

### Statistical analyses

Symptoms were compared between patients who reported taking MDL because of their MPN and patients who reported no employment change using a regression model adjusting for the type of MPN. The association between functional status and having taken MDL versus having no employment change due to MPN was assessed using a Cochran-Mantel-Haenszel test stratified by type of MPN. All other data, including patient demographics and disease characteristics, were summarized using descriptive statistics as appropriate.

## Results

### Patient demographics and disease characteristics

Out of 904 patients who participated in the Living with MPNs survey, 592 were employed at the time of MPN diagnosis. Among employed patients, 147 (24.8%) reported going on ≥ 1 MDL because of their MPN (MF, 37.9%; PV, 22.2%; ET, 15.3%) and 293 (49.5%) reported no employment changes resulting from their MPN. The remaining 152 employed patients (25.7%) reported other non-MDL changes in employment status (e.g., early retirement, work hour reduction) and were excluded from this analysis. Patients who took MDL had a mean (range) age of 52.3 (24–70) years. Most patients (84.4%) who took MDL were employed full-time at the time of MPN diagnosis. Patient demographics and MPN characteristics, including disease duration, were generally similar between patients who went on MDL because of their MPN and those who reported no changes in employment status, with the exception of history of thrombotic event (TE), in which patients who went on MDL were roughly twice as likely to have experienced ≥ 1 prior TE than those with no employment change (31.3% versus 15.4%, respectively; Table 1). Among patients with a history of TE, 46 patients (35.9%) reported taking MDL, whereas 101 patients (21.8%) without a history of TE took MDL.

**Table 1:** Patient demographics and disease characteristics

	Went on MDL				No employment Change (*n* = 293)
	MF (*n* = 66)	PV (*n* = 55)	ET (*n* = 26)	All MPNs (*n* = 147)	
Mean (SD) age at diagnosis (years)	51.4 (9.8)	42.2 (11.4)	38.7 (12.2)	45.7 (12.0)	48.3 (10.5)
Women, *n* (%)	38 (57.6)	36 (65.5)	23 (88.5)	97 (66.0)	207 (70.6)
Employment status at diagnosis, *n* (%)					
Full-time (≥ 40 h/week)	56 (84.8)	47 (85.5)	21 (80.8)	124 (84.4)	250 (85.3)
Part-time (< 40 h/week)	10 (15.2)	8 (14.5)	5 (19.2)	23 (15.6)	43 (14.7)
Mean (SD) disease duration (years)	5.4 (5.6)	8.3 (6.7)	6.0 (6.5)	6.6 (6.3)	5.9 (6.2)
History of TE, *n* (%)	15 (22.7)	20 (36.4)	11 (42.3)	46 (31.3)	45 (15.4)

ET essential thrombocythemia, MDL medical disability leave, MF myelofibrosis, MPN myeloproliferative neoplasm, PV polycythemia vera, TE thrombotic event

### Medical disability leave

Among the 147 patients who reported going on MDL because of their MPN, the mean duration from diagnosis to first leave was 2.3 years, with 44 patients (29.9%) taking ≥ 2 MDLs (Table 2). For nearly half of patients who went on MDL (48.3%), the most recent leave was long-term. Most patients who took MDL (62.6%) did not return to work; among patients who did return to work following MDL (37.4%), the mean duration of leave was 4.3 months. Among the three MPN diseases, patients with a diagnosis of MF had the highest rates of long-term leave for the most recent MDL (57.6%) and highest rates of not returning to work (77.3%). Nearly half of all patients who took MDL (46.3%) reported receiving social security while on MDL. Most patients (85.5%) who returned to work following MDL reported receiving a similar salary to that received before the leave; 10.9% reported a decrease in salary after returning to work.

**Table 2:** Medical disability leave due to MPN among employed patients

	MF (*n* = 66)	PV (*n* = 55)	ET (*n* = 26)	All MPNs (*n* = 147)
Mean (SD) from time of diagnosis to first MDL (years)	2.2 (4.5)	2.6 (4.3)	1.8 (3.1)	2.3 (4.2)
≥ 2 MDLs,^a^*n* (%)	13 (19.7)	18 (32.7)	13 (50.0)	44 (29.9)
Most recent MDL, *n* (%)				
Type: long term^b^	38 (57.6)	24 (43.6)	9 (34.6)	71 (48.3)
Did not return to work^c^	51 (77.3)	28 (50.9)	13 (50.0)	92 (62.6)
Returned to work	15 (22.7)	27 (49.1)	13 (50.0)	55 (37.4)
Mean (SD) length of leave (months)	5.7 (5.9)	4.6 (7.9)	2.2 (2.0)	4.3 (6.5)
Received social security, *n* (%)	42 (63.6)	21 (38.2)	5 (19.2)	68 (46.3)

ET essential thrombocythemia, MDL medical disability leave, MF myelofibrosis, MPN myeloproliferative neoplasm, PV polycythemia vera

^a^The number of multiple disability leaves may have been underestimated; very newly diagnosed patients were not followed long enough for adequate assessments

^b^Long-term leave was defined at the discretion of patients

^c^The number of patients who did not return to work may have been underestimated; data were truncated for those whose most recent leave was very close to the end of the observational window

### Symptom burden

**Fig. 1:**
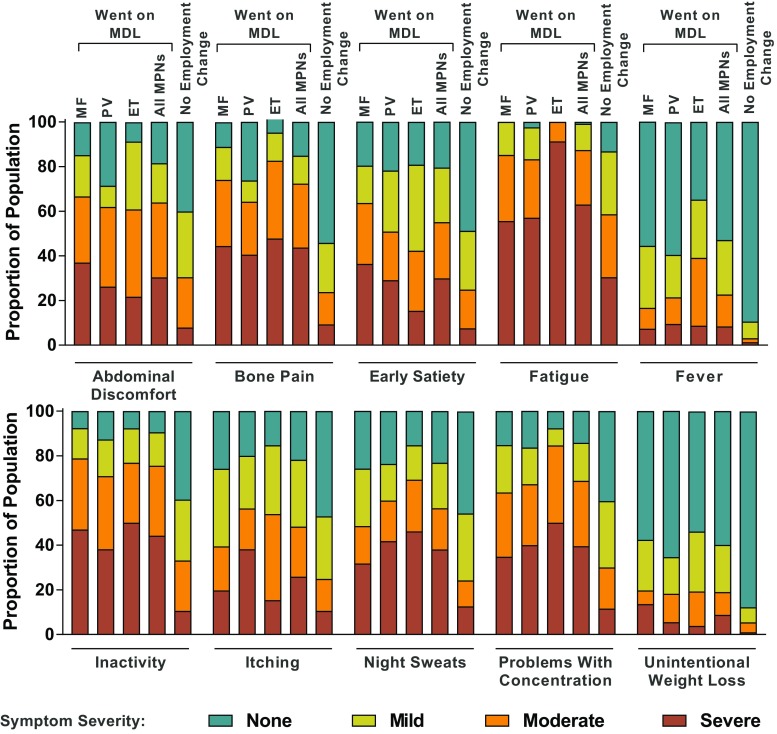
Patient-reported symptom severity. ET essential thrombocythemia, MDL medical disability leave, MF myelofibrosis, MPN myeloproliferative neoplasm, PV polycythemia vera

Patients who went on MDL because of their MPNs generally experienced higher frequency and a more severe degree of all 10 symptoms in the MPN-SAF compared with patients who reported no change in employment status (Fig. 1). Patients who took MDL had a mean MPN-SAF TSS more than double that of patients who had no employment change (42.9 versus 20.1, respectively; *P* < 0.001; Table 3). The mean MPN-SAF TSS in patients who took MDL was similar across MPN diagnoses. Among patients who took MDL, fatigue, inactivity, and bone pain were the most severe symptoms, with mean symptom scores of 7.1, 5.6, and 5.4, respectively, and were “severe” in 63.0%, 44.2%, and 43.7% of patients. In contrast, among patients who reported no employment change because of their MPN, the mean symptom scores for fatigue, inactivity, and bone pain were 4.4, 2.4, and 1.9, respectively, and these symptoms were severe in 30.4%, 10.6%, and 9.3% of patients. The mean MPN-SAF TSS as well as the scores for each of the 10 individual symptoms were significantly worse in patients who took MDL versus those who did not.

**Table 3:** Symptom burden among employed patients who took MDL versus those who had no employment change

Mean (SD) MPN-SAF score	Went on MDL				No employment change (*n* = 293)	International MPN survey (*N* = 1425) [3]
	MF (*n* = 66)	PV (*n* = 55)	ET (*n* = 26)	All MPNs* (*n* = 147)		
MPN-SAF TSS	43.2 (22.3)	41.7 (23.8)	44.8 (17.9)	42.9 (22.1)	20.1 (16.6)	21.2 (16.3)
Individual symptoms^a^						
Fatigue	6.9 (2.6)	6.9 (3.0)	8.3 (1.3)	7.1 (2.6)	4.4 (3.1)	4.4 (2.8)
Inactivity	6.0 (3.1)	5.1 (3.2)	5.6 (3.1)	5.6 (3.2)	2.4 (2.7)	2.4 (2.7)
Bone pain	5.4 (3.2)	5.0 (3.7)	5.9 (2.5)	5.4 (3.3)	1.9 (2.6)	1.9 (2.8)
Problems with concentration	4.8 (3.3)	5.1 (3.3)	6.0 (2.8)	5.1 (3.2)	2.4 (2.8)	2.5 (2.8)
Night sweats	4.2 (3.7)	4.9 (3.8)	5.2 (3.2)	4.6 (3.6)	2.2 (2.8)	2.1 (2.8)
Abdominal discomfort	4.9 (3.2)	4.2 (3.3)	4.3 (2.6)	4.6 (3.1)	2.2 (2.5)	1.8 (2.4)
Early satiety	4.7 (3.3)	3.9 (3.1)	3.3 (2.5)	4.2 (3.1)	1.9 (2.5)	2.5 (2.7)
Itching	3.4 (3.2)	4.6 (3.5)	3.8 (2.5)	3.9 (3.2)	2.1 (2.7)	2.2 (2.9)
Fever	1.6 (2.5)	1.6 (2.5)	2.6 (2.6)	1.8 (2.5)	0.3 (1.2)	0.4 (1.2)
Unintentional weight loss	1.9 (3.1)	1.5 (2.6)	1.5 (2.3)	1.7 (2.8)	0.4 (1.3)	1.1 (2.2)

ET essential thrombocythemia, MDL medical disability leave, MF myelofibrosis, MPN myeloproliferative neoplasm, MPN-SAF TSS MPN Symptom Assessment Form Total Symptom Score, PV polycythemia vera

^a^Includes patients who provided responses; sample size ranges: MF, *n* = 54–66; PV, *n* = 42–55; ET, *n* = 23–26

*P < 0.001 versus no employment change group for MPN-SAF TSS and all individual symptoms using a regression analysis adjusted for type of MPN

### Functional impairment

**Fig. 2:**
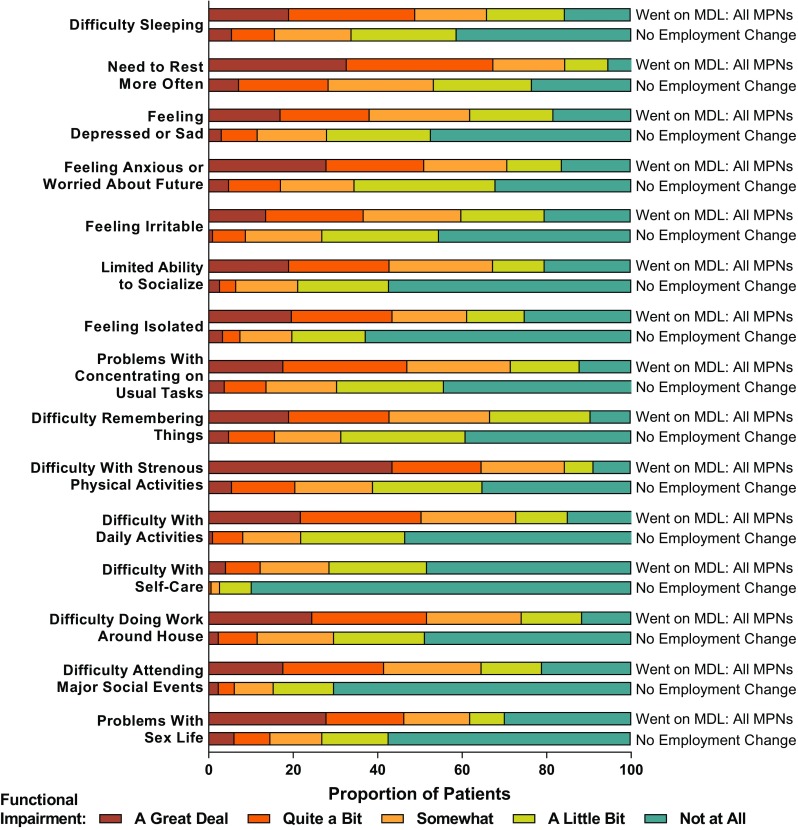
Patient-reported functional impairment. MDL medical disability leave, MPN myeloproliferative neoplasm

All aspects of functional impairments assessed in this analysis were more common and more severe among patients who took MDL than those who reported no change in employment (Fig. 2). Prevalent functional impairments among patients who took MDL compared with those who reported no employment change included difficulty with strenuous physical activities (91.2% versus 64.8%; *P* < 0.001), difficulty remembering things (90.5% versus 60.8%; *P* < 0.001), difficulty doing work around the house (88.4% versus 51.2%; *P* < 0.001), difficulty sleeping (84.4% versus 58.7%; *P* < 0.001), feeling anxious or worried about the future (83.7% versus 67.9%; *P* = 0.002), and feeling depressed or sad (81.6% versus 52.6%; *P* < 0.001).

## Discussion

In this analysis, approximately one in four patients who were employed at the time of MPN diagnosis went on MDL as a result of their MPN. Nearly half of these patients took long-term leave, with half receiving social security during leave. Most patients who went on MDL did not return to the workforce.

Patients with MPNs face a number of burdensome symptoms, which are associated with reductions in overall survival [9] and quality of life [3, 4, 6]. Physical and mental symptom burden can interfere with one’s ability to work across a wide spectrum of disorders and physiological states [10–12]. Studies of patients with various cancers demonstrate an inverse relationship between symptom burden and employment [13–15]. In the current study, the symptom burden of patients who reported no employment change was comparable to that of 1425 patients with MPNs in a prospective international survey (mean MPN-SAF TSS of 20.1 versus 21.2) [3]. However, symptoms were more frequent and severe in patients with MPNs who reported taking MDL (e.g., MPN-SAF TSS, 42.9) versus those with no change in employment status. Findings from a separate analysis of the effects of MPNs on work productivity in respondents of the Living with MPNs survey demonstrated a significant negative correlation between symptom burden and work productivity [7]. Studies in other symptomatic diseases, such as arthritis, psychiatric disorders, and gastrointestinal diseases, also show significantly higher numbers of sickness absences compared with the general population [16, 17]. These findings underscore the prominent role that symptoms play in affecting the functional ability for everyday life and work among patients with MPNs. As fatigue is consistently reported as having the highest symptom burden in patients with MPNs and was worse among patients taking MDL, fatigue symptom burden could be useful in identifying patients at risk of taking MDL and for implementing treatments and navigating management strategies.

Consistent with the increased symptom burden, patients who took MDL were more likely than those who did not to report functional impairment across physical, mental, and social domains, such as difficulty sleeping, feeling depressed, difficulty remembering, difficulty with self-care, and difficulty attending major social events. Functional impairments reported by patients with MPNs may interfere with work ability as well as activities of daily living. For instance, 55.1% of respondents in a separate analysis of the Living with MPNs survey reported receiving help from a caregiver, and among those who received assistance, 23.9% reported receiving caregiver help “often” [8]. The most common types of caregiver assistance provided to patients were homemaking (77.7%), companionship (57.4%), and transportation (55.2%). Symptom burden and functional impairments experienced by patients with MPNs can also have a negative impact on social domains. Results of the MPN Landmark survey demonstrated that many patients with MPNs reported interference with family or social life and canceling planned activities due to their disease [4]. In the current study, patients who took MDL were generally more likely to report limited ability to socialize, feeling isolated, difficulty in attending major social events, and problems with sex life versus those who did not take MDL.

In this study, 63% of patients who took MDL did not return to work, with the highest rates observed among patients with MF (77%). This rate may have been influenced by early retirement status, as patients with MF were generally older (mean age at diagnosis, 51 years) compared with patients with PV (42 years) or ET (39 years). Measures to improve patients’ ability to return to work—such as flexible work hours, temporary work-hour reductions, and options for remote access—may increase the rate of return to work following MDL among patients with MPNs. The rate of return to work in patients with MPNs in the current study (37%) was within the range reported in a literature review of patients with various cancers (mean across all studies, 62%; range, 30–93%) [14]. However, it is important to note that a variety of factors play a role in determining whether or not a patient with cancer is able to return to work, making it difficult to compare between studies and cancer settings. Longitudinal examination of other chronic illnesses reported substantial rates of “not working” status in patients diagnosed with several diseases, including heart disease (61%), arthritis (45%), diabetes (44%), back pain (37%), and hypertension (30%) [18]. High rates of MDL and the inability of many patients with chronic illnesses to return to the workforce may have significant societal implications. For instance, employers are faced with work interruption and lost productivity [6, 7], and patients and their families may experience financial hardship due to limitations in wages or salaries and reduced income while on MDL [7]. Analyses of claims data demonstrated that patients with select cancers faced increased indirect personal costs compared with controls resulting from absenteeism and short-term disability [19]. Additionally, a longitudinal study of income loss among cancer survivors demonstrated that labor market earnings were reduced by up to 40% and total family income reduced by 20% at 2 years following a cancer diagnosis, with more pronounced losses observed among men [20]. Finally, society as a whole is affected by the increased burden placed on social safety net programs such as social security.

Limitations to this study included those inherent to self-reported patient surveys. For example, these patient-reported findings were not validated with data from medical records or feedback from treating physicians. Limited data on clinical characteristics of the disease, including risk level and disease stage, were available for this analysis. Although not assessed in depth in this analysis, history of TE was more prevalent among patients who took MDL versus those with no employment change, and may have contributed to the need for MDL. Furthermore, selection bias regarding participation in the online survey as well as recall bias of the respondents may have impacted results of this study. Finally, financial hardship resulting from taking disability leave and not returning to work was not assessed in this analysis and should be addressed in future studies.

## Conclusion

Approximately one in four employed patients took at least one MDL due to their MPN disease. Patients with a history of TE were over 50% more likely to have taken MDL compared to patients without a history of TE. Patients with MPNs who reported taking MDL because of their MPN had more frequent and severe MPN-related symptoms and greater functional impairments compared with patients who reported no employment change. Most patients who went on MDL did not return to the workforce. Future studies should explore whether effective prevention of TE and management of MPN-related symptoms influences the need for forced medical leave among patients with high symptom burden.
